# 

**Published:** 1876-02

**Authors:** 


### EDITORIAL. ETC.

---

*Alumni Meeting.*—All graduates of the Baltimore College of Dental Surgery are respectfully invited to attend a meeting to be held in the College Building, Corner Lexington and Eutaw Streets, Baltimore City, on Wednesday, March 8th, 1876, at 12 o'clock noon, for the purpose of organizing an "Alumni Association." The Annual Commencement of this College will be held at the Academy of Music, on Friday evening, March 10th, 1876.

JOHN W. FARMER, M. D., D. D. S.,
GEORGE F. KEESEE, D. D. S.
*For Alumni.*

*Southern Dental Association.*—The Eighth annual meeting of this Association will be held in Nashville, Tennessee, on the 11th of April, 1876, being the second Tuesday of the month. All dentists of good standing in the profession are cordially invited to be present. Dr. Wm. T. Arrington, of Memphis, Tenn., is the President of this Association, and Dr. J. Hall Moore, of Richmond, Va., the Treasurer. Dr. Jas. F. Thompson, of Fredericksburg, Va., the Secretary, will furnish any information desired concerning the annual meeting.

The April meeting will be the third held in the Mississippi Valley during the past three years, and it is now time to choose another point further East and South. After the St. Louis meeting came the one at Memphis, in the same neighborhood, and the one now to be held at Nashville, is still in a triangle of the same section of country as the two preceding places. To have chosen a point East for the present meeting, would have afforded an opportunity for many of the members residing in Atlantic States to participate without being compelled to go so far from home, especially as this is the third time they have been called upon to do so within the past three years. We trust that a sufficient number of the members from the Atlantic States may attend the Nashville meeting to accomplish this object. Such a change of location will certainly prove beneficial to the Association.

### TO READERS AND CORRESPONDENTS.

Communications intended for publication, and exchange journals and papers, should be addressed to the EDITOR OF THE AMERICAN JOURNAL OF DENTAL SCIENCE, 259 N. Eutaw St., Baltimore, Md. All letters relating to the business of the journal, or containing cash remittances, to be directed to Messrs. SNOWDEN & COWMAN. Articles for publication should be received by the editor at least three weeks previous to the first day of the month on which the number in which it is designed for them to appear, is issued.

### CONTENTS:

### ORIGINAL COMMUNICATIONS.

### MONTHLY SUMMARY.

Treatment of Neuralgia................................ 473
Treatment of Facial Neuralgia......................... 474
Antidote to Carbolic Acid............................. 474
Plastic Operation for Lower Lip....................... 475
Borate of Soda Gargles................................ 476
Quinine Gargle in Sore Throat......................... 476
Glycosuria............................................ 477
New Remedy for Burns.................................. 477
Making a New Nose out of a Finger..................... 477
Application to Burns.................................. 478
Mode of Administering Ether........................... 478
Chloral for Ulcers.................................... 479
Antiseptic Properties of Salicylic and Benzoic Acids.. 480
Glass Cement.......................................... 480

☞ The American Journal of Dental Science will contain fifty pages of reading matter in each number, making a volume of not less than

*Six Hundred* pages

EXCLUSIVE OF ADVERTISEMENTS.

THE

### AMERICAN JOURNAL

OF

### DENTAL SCIENCE.

F. J. S. GORGAS, M. D., D. D. S., Editor.

The *Journal* is issued on the first of each month and contains not less than *fifty* pages of reading matter in each number, exclusive of advertisements, making a volume of *six hundred* pages per annum.

In each number will be found Original Communications, Correspondence, Selected Articles, Monthly Summary, Bibliographical Notices and Editorial Matter, and every effort will be exerted to render the *Journal* worthy of the patronage of the Dental profession.

A generous co-operation is respectfully solicited from the members of the profession; and as the *Journal* has now a printing office of its own, which materially lessens the cost of its publication, it will be issued at the reduced subscription price of

**$2.50 Per Annum in Advance.**

Communications are respectfully solicited on all subjects pertaining to the practice of dentistry, as well as reports of cases occurring in dental practice.

### RATES OF ADVERTISING.

| | 1 MO. | 2 MO. | 3 MO. | 6 MO. | 12 MO. |
|---|---|---|---|---|---|
| 1 PAGE. | $15 00 | $22 50 | $30 00 | $50 00 | $100 00 |
| ½ " | 10 00 | 15 00 | 20 00 | 30 00 | 50 00 |
| ¼ " | 7 00 | 11 00 | 15 00 | 20 00 | 30 00 |
| ⅛ " | 5 00 | 8 00 | 11 00 | 15 00 | 20 00 |

All original communications designed for publication, works for review, new instruments for notice, exchanges, &c., should be directed to the editor, 259 N. Eutaw St., Baltimore, Md.

All advertisements and subscriptions should be addressed to

SNOWDEN & COWMAN,

*Publishers of the Am. Journal of Dental Science.*

86 W. FAYETTE ST. BET. CHARLES & LIBERTY STS.,

BALTIMORE, MD.

